# Bidirectional crosstalk between the gut microbiota and cellular compartments of brain: Implications for neurodevelopmental and neuropsychiatric disorders

**DOI:** 10.1038/s41398-025-03504-2

**Published:** 2025-08-13

**Authors:** Seyedeh Marziyeh Jabbari Shiadeh, Wing Ki Chan, Sofia Rasmusson, Noor Hassan, Sâmia Joca, Lars Westberg, Anders Elfvin, Carina Mallard, Maryam Ardalan

**Affiliations:** 1 Department of Physiology, Institute of Neuroscience and Physiology, Sahlgrenska Academy, University of Gothenburg, Gothenburg, Sweden; 2 Department of Pharmacology, Sahlgrenska Academy, University of Gothenburg, Gothenburg, Sweden; 3 Department of Biomedicine, Aarhus University, Aarhus, Denmark; 4 Department of Pediatrics, Institution of Clinical Sciences, Sahlgrenska Academy, University of Gothenburg, Gothenburg, Sweden; 5 Region Västra Götaland, Department of Pediatrics, The Queen Silvia Children’s Hospital, Sahlgrenska University Hospital, Gothenburg, Sweden; 6 Department of Clinical Medicine, Translational Neuropsychiatry Unit, Aarhus University, Aarhus, Denmark

**Keywords:** Molecular neuroscience, Physiology, Autism spectrum disorders

## Abstract

The gut-brain axis serves as a crucial communication pathway, with microbial metabolites such as short-chain fatty acids (SCFAs) playing a central role in regulating neuroinflammation and maintaining neuronal health. The gut microbiota’s impact on neurodevelopment is highlighted, particularly its relevance to autism, anxiety, and other psychiatric conditions. In this review, we explored the intricate relationship between the gut microbiota (GM) and the central nervous system (CNS), emphasizing the bidirectional communication that forms the gut-brain axis. Associations between specific gut microbiota and neurodegenerative diseases are explored, focusing on the role of certain bacteria in processes such as amyloid aggregation and neuroinflammation in Alzheimer’s disease (AD) and Parkinson’s disease (PD). The potential for therapeutic modulation of the gut microbiota is discussed, with a focus on dietary interventions and probiotics as strategies to improve outcomes in neurodegenerative diseases by restoring gut health. We concluded by emphasizing the significance of understanding the gut-brain connection and calls for further research to investigate therapeutic approaches targeting the gut microbiome for brain health.

## Introduction

The human gut harbors a diverse array of microorganisms, encompassing various taxa, such as bacteria, viruses, protozoa, and fungi [1]. The human genome comprises around 23,000 protein-coding genes, whereas the human microbiome contains over three million functionally diverse genes that produce metabolites vital for host functions such as immune regulation, nutrient processing, and gut–brain signaling [2]. Among the microbiota, *Bacteroidetes* and *Firmicutes* are predominant components of the typical gut flora, collectively representing 90% of the bacterial phyla [3–5]. In vivo studies have demonstrated that a well-balanced gut microbiota is critical not only for maintaining intestinal barrier integrity but also for supporting bidirectional communication along the gut–brain axis [6]. Conversely, microbiota dysbiosis an imbalance in microbial composition is associated with disturbances in brain development, behavior, and mental health, as it disrupts signaling pathways that interconnect the gut, immune system, and central nervous system (CNS) [7]. Recent findings indicated the impact of gut microbes on brain-resident cells, including microglia and astrocytes. For example, fecal microbiota transplantation (FMT) has been shown to reduce neuroinflammatory responses in regions such as the substantia nigra and to suppress Toll-like receptor-4 (TLR4)/TNF-α pathway activity in both the gut and brain [8]. On the basis of this interaction, GI dysfunction can be a prominent comorbidity in neurodevelopmental disorders such as autism [9]. Several studies have reported altered gut microbiota profiles in individuals with autism spectrum disorder (ASD), including increased abundances of genera such as *Clostridium*, *Ruminococcus, Sutterella*, and *Lactobacillus*, and decreased levels of *Bifidobacterium*, *Akkermansia*, *Blautia*, and *Prevotella*. For instance, Wang et al. specifically identified a higher abundance of *Sutterella spp*. and *Ruminococcus torques* in fecal samples of children with ASD compared to their typically developing siblings and unrelated community controls, though the study did not specify whether these groups were matched for age or sex. This highlights the importance of clearly defining control groups in microbiome studies, given the variability in what constitutes a “healthy” gut microbiome [10–15].

The influence of the intestinal microbiota on brain‒gut interactions starts early in life and continues into old age [16]. Therefore, neurodevelopmental and neuropsychiatric disorders may be treated by developing new interventions on the basis of advancements in neuroscience–microbiome research. Indeed, understanding the dynamic interactions between the gut microbiota and brain cells holds great promise for developing novel therapeutic strategies for a wide range of neurological conditions, including depression, anxiety, autism, and neurodegenerative diseases. In this review, we aim to discuss current knowledge on the role of the gut microbiome in modulating brain function, with a focus on cellular interactions between gut-derived signals and brain-resident cells including neuronal and non-neuronal cells. We explore how the microbiota influences these cells under both physiological and pathological conditions and examine the implications for neurodevelopmental, neuropsychiatric and neurodegenerative disorders. By highlighting recent advances in microbiome-brain research, we aim to identify emerging therapeutic opportunities and outline future directions for the development of microbiome-based interventions in brain disorders.

## Early-life gut microbial composition

The symbiotic relationship between infants and their gut microbiome begins at birth and evolves throughout the formative years of infancy. This relationship is particularly significant during the early postnatal phase, which aligns with the critical period of early brain development and the initial establishment and growth of the gut microbiome. The composition of the gut microbiome undergoes significant transformations from birth to adulthood, influenced by the ongoing expansion and diversification of dietary intake [17]. Environmental stressors during this period, such as food insecurity and infections, can disturb the optimal development of the microbiome, with potential long-term effects that may persist throughout an individual’s life and even across generations [18]. The impact of the early-life microbiome on a child’s growth may depend on both its composition and functionality. For example, the inheritance of genes related to bile acid metabolism and starch consumption, which can vary among individuals, is likely crucial for nutrient absorption and, consequently, in the child’s early growth [18, 19].

The GM plays an important role in host health by producing a variety of metabolites through the fermentation of dietary components. These compounds, produced through microbial metabolism of dietary and endogenous substrates, include short-chain fatty acids (SCFAs), bile acid derivatives, tryptophan catabolites, and phenolic compounds, among others [20]. SCFAs are free fatty acids with fewer than six carbons, such as propionic acid (C3), butyric acid (C4), valeric acid (C5), acetic acid (C2), and formic acid (C1) [21]. SCFAs are primarily produced when undigested dietary fibers reach the colon and are fermented by specific gut bacteria, which use these fibers as substrates [22]. Compared with adults, newborns fed human milk have fecal SCFA levels that are characterized by comparatively higher proportions of acetate, lower proportions of propionate, and nearly no butyrate. Additionally, lactate is more frequently found in the feces of newborns because lactate-utilizing bacteria convert it right away. The predominance of *Bifidobacteria* and *Lactobacilli* is reflected in the high levels of acetate and lactate found in newborns given human milk [23]. Especially *Bifidobacteria* metabolize human milk oligosaccharides (HMOs) to produce mainly acetate and formate, with lesser amounts of lactate [24]. Similarly, *Lactobacilli* produce lactate, helping to create an acidic environment in the infant gut that inhibits pathogenic bacteria and supports overall gut health [25]. However, the slow development of butyrate-producing bacteria within *Firmicutes*, a bacterial group that is less prevalent in colicky infants, might result in the prioritization of lactate and acetate utilization over higher levels of potentially harmful *Proteobacteria* members [26]. Variations in the GM during the early stages of life impact the developing brain by modifying synaptic plasticity, which results in long-term abnormal changes to synaptic transmission [27]. However, further research is needed to fully comprehend how the composition of the GM during infancy and its reciprocal interactions with the brain might affect vulnerability to neurological and psychiatric disorders. In addition to gut-to-brain communication, increasing evidence highlights the importance of brain-to-gut signaling in shaping the gut microbiota. This top-down regulation occurs primarily through the autonomic nervous system, hypothalamic–pituitary–adrenal (HPA) axis, and neuroimmune pathways [28]. For example, psychological stress or altered neurodevelopmental states can modulate gut physiology by changing gut motility, mucus secretion, and intestinal barrier function, which in turn can affect microbial colonization and diversity. Stress-induced activation of the HPA axis leads to the release of glucocorticoids, which can suppress immune function and shift the microbial composition toward a more pro-inflammatory state [29, 30]. Furthermore, neural inputs via the vagus nerve can influence intestinal immune responses and microbial dynamics. These brain-driven effects may be particularly relevant in neurodevelopmental disorders such as autism, where altered sensory processing or stress responses could indirectly contribute to gut dysbiosis.

## Microbiota and metabolomics

Microbial populations play a substantial role in altering a significant portion of the environmental impact on human health and susceptibility to illness [31]. Research indicates that the human gut microbiota comprises more than 1200 distinct bacterial species, with each individual harboring a unique combination of at least 160 species in their gut [32]. Over 104 microorganisms, including 300–3000 different species, constitute the microbiota that lives in the gut [33]. The gut‒brain axis involves bidirectional communication between the brain and gut microbiota irrespective of the existence of the blood‒brain barrier (BBB) [34]. Especially during pregnancy, when bacterial metabolites can be transferred from the mother to the fetus, alterations in the mother’s gut microbiome might result in the suppression of gut tight junction gene expression [35]. This could expose the fetus to biomacromolecules and microorganisms that trigger neuroinflammatory mechanisms [19, 36, 37]. Under stable conditions, monocarboxylate transporters facilitate the passage of all three primary SCFAs across the BBB, which are detected in the cerebrospinal fluid (CSF) of humans [38, 39]. On the other hand, SCFAs have the potential to travel from the intestinal mucosa into the systemic circulation, affecting the number of microglia and astrocytes [40]. Given their role in restoring microglial density and morphology in germ-free mice, SCFAs may contribute to neuroprotection by supporting normal microglial function. This, in turn, has been linked to cognitive benefits, including improved memory and the restoration of cognitive function [41, 42]. Additionally, butyrate, a type of SCFA, also promotes glial cell-derived neurotrophic factor (GDNF) expression in astrocytes, which are essential for regulating neuronal growth, survival, and synaptic differentiation [43].

SCFAs, bile acids, trimethylamine N-oxide, branched-chain amino acids (BCAA) and indole derivatives are some of the types of metabolites produced by the microbiota [44–46]. The liver produces primary bile acids such as chenodeoxycholic and cholic acid, whereas the colonic gut-resident populations generate secondary bile acids, including lithocholic acid and deoxycholic acid [47]. Moreover, gut bacteria can also control the levels of various neurotransmitters locally and in the brain, including glutamate, Gamma Aminobutyric Acid (GABA). Interestingly, dysregulated levels of these neurotransmitters in the brain are associated with the neurobiology of neurological and mental disorders [48]. Notably, 90% of serotonin is generated in the gut and can interfere with mood, sleep and behavior, among other brain functions [49]. GABA, the main inhibitory neurotransmitter in the brain, can also be produced by human microbiota communities, such as *Lactobacillus brevis* and *Bifidobacterium dentium* [50, 51]. Since GABA produced in the gut is unable to cross the BBB, it can affect the brain indirectly, affecting the activity of the vagus nerve or the enteric nervous system (ENS). Other microbiota metabolites, such as acetate in the colon, enter the bloodstream, cross the BBB, increase hypothalamic acetate levels, and feed into the GABA neuroglial cycle to increase central GABA production, preferentially in the hypothalamus [52]. Therefore, the GM has the capacity to alter neurotransmitter balance and brain excitability [53], which are central mechanisms in the development of neurological and mental conditions, such as depression, anxiety, autism, and schizophrenia [54, 55]. Among SCFAs, propionic acid (PPA) is proposed to be the link between food, GM composition and brain function by regulating metabolic changes, inflammatory pathways and intracellular potassium levels, but the precise mechanisms are not entirely clear [56–60]. While the precise mechanisms remain unclear, growing evidence suggests that SCFAs, including PPA, modulate brain health not only through direct neurochemical effects but also by shaping the immune system, particularly through the differentiation and activity of regulatory T cells. These immune cells influence neuroinflammatory responses, and SCFA-mediated signaling has been shown to affect microglial function and T cell trafficking to the brain, thereby connecting gut-derived metabolites with central nervous system outcomes. Recent studies have shown that SCFAs, particularly butyrate, acetate, and propionate, produced by gut microbial fermentation of dietary fibers, play key roles in modulating the immune system. These SCFAs influence the differentiation and function of regulatory T cells (Tregs), which are essential for maintaining immune homeostasis and suppressing neuroinflammation [61]. Erny et al. demonstrated that Gut microbiota metabolites can prime CD4⁺ T cells to acquire encephalitogenic properties outside the brain, such as in intestinal lymphoid tissues or local draining lymph nodes [41]. Moreover, SCFAs such as acetate, propionate, and butyrate, produced by gut microbial fermentation of dietary fibers, have been shown to regulate the differentiation of naive T cells into various subsets, including regulatory T cells (Tregs) and effector T cells. This modulation occurs through both direct and indirect mechanisms, impacting immune homeostasis and inflammation [21]. Therefore, understanding how intestinal microbial metabolites affect the function of brain cells individually, both in healthy individuals and in individuals with neurobehavioral abnormalities, is important to better characterize the role of the GM in disease vulnerability.

## Interaction between the gut microbiota and brain cells

The ENS, a tightly interconnected network of enteric glial cells and neurons located within the intestinal wall, regulates the motility, permeability, integrity, and immune function of the gastrointestinal tract [62–64]. Recent preclinical research has revealed a regulatory role of the gut microbiome on microglial morphology and function, as well as other brain cells that can cause several brain disorders [65].

### Neuronal and interneuronal cells

A number of brain areas, including the amygdala and hippocampus, show age-dependent reliance on the GM for typical neuronal growth, as neurons proliferate more rapidly in the dorsal hippocampus during early childhood [66]. Furthermore, there are differences in neuronal plasticity between the dorsal and ventral hippocampi. In the basal lateral amygdala (BLA), both aspiny interneurons and pyramidal neurons display dendritic hypertrophy along with an increased number of thin, mushroom and stubby spines. Conversely, in the ventral hippocampus of germ-free mice, pyramidal neurons exhibit shorter lengths, less branching, and a lower number of mushroom and stubby spines [66]. The colonic microbiota has also been shown to support the maintenance of nitrergic neurons and the ENS in adult mice by stimulating intestinal neurogenesis through Toll-like receptor 2 (TLR2) activation [67]. Nitrergic neurons are a type of neuron that utilizes nitric oxide (NO) as a neurotransmitter. Importantly, TLR2 activation in this context occurs on enteric neurons and enteric neural precursor cells, suggesting a direct effect of microbiota-derived signals on the ENS. Studies have shown that microbiota-derived components, such as peptidoglycan and lipoproteins, can engage TLR2 signaling pathways directly on these enteric neural populations. This activation of intestinal neurogenesis through TLR2 supports the maintenance of these neurons, which are essential for the proper functioning of the enteric nervous system in adult mice [68]. In particular, Brun et al. (2013) demonstrated that TLR2 knockout mice exhibited a reduction in enteric neuron number and impaired gastrointestinal motility, highlighting the receptor’s essential role in ENS homeostasis [69].

*Escherichia, Parabacteroides* and *Bacteroides* are three main bacterial species that actively express GABA-producing neuropeptides, including calcitonin gene-related peptide, substance P, neuropeptide Y (NPY), somatostatin, vasoactive intestinal polypeptide and corticotropin releasing factor, in human stool samples [70]. Owing to their shared receptors on cell membranes, typically G-protein-coupled receptors (GPCRs), neuropeptides and gut metabolites often induce similar biological effects. Notably, this convergence is evident in members of the pancreatic polypeptide (PP-fold) family of peptides, namely, peptide YY (PYY) and NPY. Within the gut–brain axis, NPY is ubiquitously expressed, whereas enteroendocrine cells serve as the primary site for PYY expression. The influence of NPY extends across various factors, including behavior, pain, inflammation, and brain function, potentially modulating how gut metabolites impact these aspects [71].

Persistent stress can change the balance of the GM, promoting harmful bacteria over beneficial bacteria, a condition known as dysbiosis. Dysbiosis is associated with aging, potentially resulting in chronic inflammation and decreased levels of SCFAs and other proneurogenic bacterial metabolites in the aged intestine [72]. The microbiota affects brain development and growth phenotypes, as shown by a reduction in the expression of neuronal markers such as neurofilament-L and NeuN, along with myelin basic protein, a myelin marker [73]. Moreover, these findings suggest a top-down (brain-to-gut) influence, possibly via neuronal or hormonal mechanisms (e.g., HPA axis). Interneurons within the ENS play a crucial role in coordinating local reflexes and modulating intestinal functions. Alterations in interneuronal signaling can disrupt these processes, leading to dysbiosis. For instance, stress-induced changes in CNS activity can affect the release of neurotransmitters and hormones, such as corticotropin-releasing factor (CRF), which in turn influence gut permeability and immune function, thereby impacting microbial composition [74].

In summary, the GM plays a crucial role in supporting neuronal growth in the CNS and ENS, with age-dependent effects on neurogenesis. Moreover, the GM influences dendritic hypertrophy, spine density, and branching patterns in specific brain regions, highlighting the role of gut microbes in shaping neuronal structure and function. The exact mechanisms linking the GM to changes in neuronal function throughout life are not well understood, and investigations of these mechanisms may provide new insights for the development of improved therapeutic strategies for brain disorders.

### Microglia

Microglia are highly specialized macrophages that make up approximately 5–15% of brain cells and are essential for maintaining brain homeostasis and CNS development [75]. Interestingly, the GM can regulate the development of innate immune cells, including macrophages, and thereby influence innate immune responses [76]. The maternal microbiota profoundly influences the development of microglia in offspring, with effects varying by sex and age [77]. Compared with those of specific pathogen-free (SPF) dams, offspring of germ-free (GF) dam’s present sex-specific changes in gene expression, increased microglial ramification and density, and altered chromatin accessibility in embryonic microglia. A lack of specific microbiota stimulates microglial proliferation in specific brain regions (hippocampus and hypothalamus) of newborn mice [78]. Germ-free mice are completely free of any microorganisms, whereas pathogen-free mice are animals that are free from specific, known harmful pathogens but may still carry non-pathogenic microorganisms. Microglia sensitivity to stimuli is influenced by the GM, leading to significant morphological and genetic variations in microglia from GF and SPF mice. These variations result in marked differences in the structural and functional development of microglia [41]. SCFAs interact with GPCRs, including GPR41 and GPR43, expressed on microglia. This activation modulates microglial inflammatory responses and cytokine production, contributing to neuroprotection [79]. For example, butyrate function as a histone deacetylase (HDAC) inhibitor, leading to changes in chromatin accessibility and transcriptional activity in microglia. This epigenetic modification plays a crucial role in maintaining microglial homeostasis and suppressing neuroinflammatory responses [80]. It also serves as an energy source for microglia by entering the tricarboxylic acid (TCA) cycle, thereby influencing their bioenergetics and functional states [81].

The Cx3cr1–Cx3cl1 pathway is a key mechanism for microglia (CX3CR1) –neuron (fractalkine (CX3CL1)) communication in the brain, and this interaction has been utilized to evaluate how microglia and neurons respond to dysbiosis during neurodevelopment [41, 82] *Lactobacillus*, a crucial component of the gut microbiome, can ameliorate social impairment in offspring and prevent autism-like behaviors by inhibiting microglial activation and premature senescence through the regulation of Cx3cr1 [83]. Conversely, the bacterial byproduct p-cresol (4-methylphenol), considered a uremic toxin, has been implicated in increasing levels of microglia-associated CD68 protein in the prefrontal cortex of mice with p-cresol sulfate-induced neuroinflammation [84, 85]. However, treatment with *Clostridium butyricum* shows promise in reducing inflammation mediated by microglia by modulating the gut‒brain axis through the production of butyrate metabolites [86]. Similarly, tryptophan, a metabolite of the gut microbiota, affects the activation of microglia via the aryl hydrocarbon receptor (AHRs) [87]. Furthermore, one of the metabolites of tryptophan, indole acetic acid, has been shown to reduce neuroinflammation in vitro in BV2 microglia stimulated with LPS through downregulation of the NF-κB pathway [88].

**Fig. 1:**
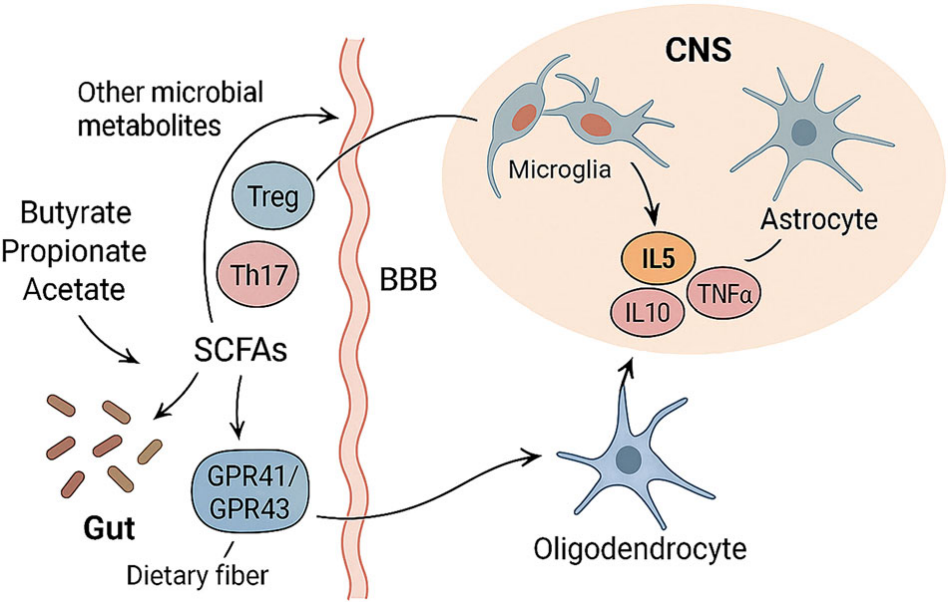
Gut microbiota-mediated modulation of CNS glial cell function. This schematic illustrates the influence of gut microbiota-derived SCFAs specifically butyrate, propionate, and acetate on central nervous system (CNS) glial cells. SCFAs are produced through the microbial fermentation of dietary fiber and act on host receptors such as GPR41 and GPR43. These metabolites modulate immune responses by promoting regulatory T cells (Tregs) and suppressing pro-inflammatory Th17 cells. Through systemic circulation and interactions with the blood brain barrier (BBB), SCFAs and other microbial metabolites impact microglia, astrocytes, and oligodendrocytes. Microglial activation subsequently regulates cytokine production (e.g., IL-10, IL-5, TNF-α), which shapes astrocyte function and potentially affects oligodendrocyte maturation and myelination, thereby influencing CNS health and disease.

While numerous studies have explored how the gut microbiota influences microglial development and function, the reverse direction, how activated microglia impact gut development and microbial composition, remains largely unexplored. Although it is biologically possible that microglial-derived signals, such as cytokines released during neuroinflammation, could modulate gut physiology via the autonomic nervous system or HPA axis, direct evidence supporting microglia-driven changes in gut development or microbiota composition is lacking. This represents a significant gap in our understanding of brain to gut communication within the gut–brain axis, particularly during early life when both the nervous and gastrointestinal systems are highly plastic. In summary, the GM significantly influences microglial development and function, affecting neuroimmune regulation through maternal microbial exposure, SCFAs, bacterial metabolites, and microglia–neuron communication pathways. These findings suggest the potential therapeutic implications of targeting the gut‒brain axis to modulate microglial function and improve brain conditions associated with dysregulated inflammation (Fig. 1).

### Astrocytes

Astrocytes, comprising 20–40% of CNS glial cells [89], are crucial for brain development, such as vascularization, BBB formation, and synaptic regulation [90]. In early postnatal development, changes in the GM appear to play a crucial role in regulating astrocyte maturation and function, with associated abnormalities in astrocytes being frequently linked to gut dysbiosis [91].

Overactive astrocytes are recognized as an important mechanism for the production of harmful neural substances that lead to dysfunction within the CNS and the onset of neurological disorders [90]. Astrocytes that express more aryl hydrocarbon receptors (AHRs) have anti-inflammatory properties because they participate in the type-I interferons (IFN-I) signaling, which restricts the entry and activity of immune cells that are detrimental to the neurological system [92]. Research indicates that 3-indoxyl sulfate (a metabolite derived from the breakdown of the amino acid tryptophan by gut bacteria) may penetrate the BBB and trigger astrocyte AHR [88]. Some GMs can shield neurons against inflammatory damage by converting tryptophan into AHR agonists [93]. Gut metabolites generated from bacteria may agonistically affect astrocyte AHR to reduce neuroinflammation through IFN-I signaling [92]. Female mice fed a high-fat and high-sugar diet presented a decrease in astrocytic density in the hypothalamus along with gut dysbiosis, which is an indication of an imbalance in the GM [94].

SCFAs originating from the gut microbiome, particularly butyrate, may have a large effect on astrocytes by modulating their mitochondrial regulation, which plays a fundamental role in astrocyte activity [91]. SCFAs differently affect gene expression in astrocytes, increasing immune-related genes like IL-22 in males and BDNF in females, highlighting sex-specific roles in neuro-immunity and anti-inflammatory pathways [95]. Experimental autoimmune encephalomyelitis (EAE) was induced in female C57BL/6 mice as a model of MS, revealing reduced SCFAs and beneficial microbes like *Lactobacillus*. SCFA supplementation boosted Trp-derived AhR ligands, activating Trp-AhR-AQP4 signaling and suppressing astrocyte activation. These results suggest SCFAs as a potential therapy for CNS inflammation [96]. Gut dysbiosis and alterations in the GM influence astrocyte activation, which in turn causes the negative emotions associated with opiate withdrawal [97]. The administration of finasteride, a known inducer of depressive-like behavior, led to increased glial fibrillary acidic protein (GFAP) astrocytes in the hippocampal dentate gyrus, along with gut dysbiosis [98] (Fig. 1). Exposing rats to chronic unpredictable mild stress (CUMS) aimed at inducing depression-like behavior resulted in the activation of hippocampal astrocytes. This activation was accompanied by the disruption of the GM, specifically altering the ratio of *Lactobacillus* to *Clostridium* [99].

While several studies indicated how the gut microbiota influences astrocyte development and reactivity, the potential for astrocytic changes in the brain to affect gut development or microbiota composition remains a largely unstudied area. To date, no direct experimental studies have demonstrated a causal link between astrocyte dysfunction and alterations in gut physiology or microbial ecology. Yet, given the central regulatory role of astrocytes in neuroinflammation, neurotransmitter homeostasis, and metabolic signaling, we speculate that astrocytic activation could influence the gut via several key pathways. Astrocytes are known to release pro-inflammatory cytokines (e.g., IL-1β, TNF-α), glutamate, ATP, and reactive oxygen species during activation, all of which can modulate neural circuits involved in autonomic regulation, including the hypothalamus and brainstem nuclei that control vagal and sympathetic outputs to the gut. Dysregulated astrocyte-neuron interactions in these regions could alter gut motility, secretion, and barrier function, thereby reshaping the microbial environment. Moreover, astrocytes contribute to glucocorticoid signaling via modulation of the hypothalamic–pituitary–adrenal (HPA) axis, suggesting that astrocyte-driven neuroendocrine changes could indirectly influence gut immune tone and epithelial maturation [100, 101].

In summary, changes in the GM have been linked to alterations in astrocyte maturation and function, with disruptions in the gut–brain axis potentially leading to abnormalities in astrocyte activity. SCFAs, particularly butyrate, are crucial mediators in this process due to their ability to cross the BBB and regulate astrocyte function via mitochondrial modulation, epigenetic modifications, and receptor-mediated signaling. The bioavailability and mechanisms of action of SCFAs suggest that these metabolites may play a protective role in neuroinflammation and neurological disorders through direct modulation of astrocyte physiology and CNS homeostasis. Moreover, there is a pressing need for studies investigating whether astrocytic reactivity or dysfunction, especially during early development or in neuroinflammatory conditions like autism spectrum disorder, can influence gut development or microbial composition.

### Oligodendrocytes

The GM is recognized as an essential factor among the elements influencing myelination in the brain and plays a pivotal role in the regulation of both myelin formation and neurobehavioral development, such as social avoidance behavior [102]. It has a direct effect on the immune system through neuroactive microbial metabolites and signaling via the vagus nerve. This leads to changes in the circulating levels of pro- and anti-inflammatory cytokines, which can have an impact on oligodendrocyte development [102, 103]. Research shows that a healthy microbiota is necessary for proper cortical myelination throughout key stages of neurodevelopment and that a dysfunctional microbiota may be a risk factor for CNS demyelination caused by inflammation [102, 104]. SCFAs can reduce oligodendrocyte precursor cell loss by inhibiting astrocyte activation via glucocorticoid-regulated kinase (SGK)1/IL-6 signaling pathway and downregulating the expression of NOD-like receptor thermal protein domain associated protein 3 (NLRP3), IL-6, CCL2, and IP-10. This finding suggests that SCFAs influence oligodendrocyte viability and function, potentially impacting myelination processes [105]. Germ-free (GF) mice also showed reduction in myelination in major gray and white matter regions and the whole brain at 4 or 12 weeks of age [106]. Furthermore, research has demonstrated that the use of bacterial metabolites might reverse the lasting impacts of neonatal ampicillin, vancomycin, and neomycin therapy on the regulation of myelin in the prefrontal cortex [107]. Because butyrate possesses HDAC inhibitory action comparable to that of valproic acid, a mood stabilizer that may suppress oligodendrocyte progenitor cell (OPC) differentiation both in vitro and in vivo, it may have a direct effect on the myelination and differentiation of OPCs [108]. Mice exhibiting social withdrawal demonstrated alterations in their gut microbiota, leading to elevated levels of p-cresol, a byproduct of microbial breakdown of dietary tyrosine. P-cresol, known for its high permeability, was observed to inhibit oligodendrocyte differentiation in vitro [109]. Additionally, during the early stages of life, when the brain is still developing and susceptible to environmental stressors that can cause a variety of disorders to appear later in life, the microbiota is essential for promoting white matter development [110] (Fig. 1). Magnetic resonance imaging (MRI), both in vivo and ex vivo, demonstrated that SPF mice displayed more gray matter than GF mice did. SPF mice at 4 weeks of age presented greater myelination in gray matter structures, such as the neocortex, hippocampus, and various major white matter tracts. Additionally, SPF males at 12 weeks of age presented increased myelination in the internal capsule [106].

In Summary, the GM influences oligodendrocyte development and function through immune signaling and metabolites like SCFAs. SCFAs promote oligodendrocyte maturation and white matter formation, while dysbiosis or microbial byproducts such as p-cresol can disrupt oligodendrocyte differentiation and impair myelination. Reduced myelination in germ-free and antibiotic-treated mice highlights the microbiota’s critical role in supporting oligodendrocyte-mediated brain development.

## Microbiota and brain disorders

Several studies have connected the gut microbiota to a range of disorders affecting the nervous system, including neurodevelopmental disorders such as autism spectrum disorders (ASDs) [111, 112] and ADHD [113–115]; neuropsychiatric disorders such as depression [116–118] and schizophrenia [119]; and neurodegenerative disorders such as Parkinson’s disease (PD) [120–122] and Alzheimer’s disease (AD) [123, 124]. Accordingly, in this section, we review the potential role of GM in the origins and development of nervous system disorders which are summarized in Table 1.

**Table 1:** Summary of gut microbiota alterations by disorder.

Disorder	References	Altered Bacteria	Microbial Metabolites/Pathways	Cellular Effects (Brain)
Autism Spectrum Disorder (ASD)	[10–12, 14, 132, 140]	↑ *Clostridium, Ruminococcus, Sutterella, Faecalibacterium, Lactobacillus*; ↓ *Bifidobacterium, Akkermansia, Blautia, Prevotella, Bacteroides, Parabacteroides*	SCFAs (↓butyrate, acetate), ↑ p-cresol; altered tryptophan metabolism; gut–brain axis signaling	Microglial activation, astrocyte reactivity; synaptic pruning and neuroinflammation
Attention-Deficit/Hyperactivity Disorder (ADHD)	[114, 115, 147]	↑ *Bacteroides ovatus*, *B. uniformis, Sutterella stercoricanis*; ↓ *Bifidobacterium* (in early infections)	SCFAs influencing BDNF and dopamine synthesis; diet–microbiota interaction	BDNF signaling; microglial and astrocyte changes inferred from SCFA influence
Depression	[116–118, 151, 154]	↓ *Blautia, Akkermansia*; ↑ *Klebsiella, Proteobacteria*	SCFA signaling via NLRP3 inflammasome; tryptophan–kynurenine imbalance	Microglial modulation, astrocytic inflammation; systemic immune activation
Schizophrenia	[119, 162, 164, 205]	↓ Butyrate producers (*Blautia, Roseburia, Coprococcus*); ↑ ASCA antibodies	Tryptophan–kynurenine pathway dysregulation; ↓ kynurenic acid, ↑ quinolinic acid	Microglial neurotoxicity, astrocyte dysfunction, behavioral effects via FMT
Alzheimer’s Disease (AD)	[123, 124, 172–174]	↑ *Escherichia/Shigella*; ↓ *Bifidobacterium, Lactobacillus*; ↑ *Curli-producing* *Enterobacteriaceae*	SCFAs; bacterial amyloids (Curli), LPS-induced inflammation	Microglial and astrocyte-mediated neuroinflammation
Parkinson’s Disease (PD)	[120–122, 199, 201, 200]	↓ *Ruminococcus, Faecalibacterium, Blautia*; ↑ *Enterococcus, Streptococcus, Escherichia, Shigella*	↓ SCFAs; ↑ LPS biosynthesis; altered BCAA and aromatic amino acid metabolism	Microglial activation, α-syn aggregation, astrocytic stress

### Neurodevelopmental disorders

Complex interactions between gene expression and prenatal and postnatal environmental factors are necessary for neurodevelopment. The acquisition and reformation of the GM occur throughout the early postnatal brain development process, which depends on the right environmental inputs during crucial times for proper development [125]. The colonization of the human microbiota starts at birth and lasts for approximately three years. During this period, it undergoes development and changes in species abundance to resemble that of an adult [126]. The GM may influence the production of neurotransmitters by impacting “de novo” synthesis or metabolic pathways related to neurotransmitters [127]. Therefore, the interactions between the GM and neurotransmitters during neurodevelopment may indicate that microorganisms play a part in the pathophysiology of a variety of neurodevelopmental disorders, including ADHD and ASD.

#### Autism spectrum disorder (ASD)

Neurobehavioral disorders known as autism spectrum disorders (ASDs) are characterized by difficulties with social communication, repetitive behaviors, and difficulties with cognitive and verbal abilities that are usually noticeable in early childhood [128]. Along with altered organic composition and an imbalanced microbiota, ASD is associated with increased gut permeability, known as “leaky gut” [129], which results in a situation where bacterial metabolites can easily pass through the intestinal barrier due to enhanced gut permeability [130]. A 20-year study of 16,440 Swedish children found significant links between early-life factors such as prenatal exposures, infections, and microbiome composition and neurodevelopmental disorders (NDDs) [131].

A reduced diversity of GM has been associated with autistic symptoms in children. A study revealed a notable reduction in bacteria with carbohydrate degradation and fermentation ability (*Coprococcus, Prevotella*, and unclassified *Veillonellaceae*) in samples from individuals with ASD compared with those from neurotypical controls [132]. Additionally, an elevated ratio of *Firmicutes/Bacteroidetes* was detected in the GM of individuals with ASD [11]. This is particularly noteworthy, as the levels of the serotonin metabolite 5-hydroxytryptamine, which is involved in anxiety, socio-affective processes and fear, can influence gut motility. Consequently, it is proposed that the GM influences intestinal motility via the serotonergic system [133].

A different study revealed a greater occurrence of *Enterococci* and *Clostridium spp*. in autistic children’s feces samples than in controls [134]. Compared with control subjects, children diagnosed with ASD had reduced proportions of *Bifidobacterium, Bacteroides, Parabacteroides* and *Akkermansia*, along with an increased proportion of *Faecalibacterium* in the total microflora detected [135]. In addition to the microbiota, metabolites such as SCFAs have been noted to be altered in the fecal samples of children diagnosed with ASD. These changes were linked to changes in *Clostridia* and *Bacteroidetes*, key producers of SCFAs [136–138]. Elevated levels of p-cresol, a metabolite primarily generated by the GM, have been observed in fecal samples from children diagnosed with ASD [84], and recently, it was discovered that urinary levels of p-cresol are associated with the severity of autism symptoms [139]. Alterations in gut microbiome composition have been implicated in ASD-related behavioral abnormalities, potentially through effects on neurodevelopmental signaling pathways. In a study using the BTBR T+ Itpr3tf/J mouse model of autism, Kratsman et al. (2016) demonstrated that sodium butyrate treatment attenuated social behavior deficits and modified the transcription of genes involved in excitatory and inhibitory neurotransmission in the frontal cortex. Importantly, the study utilized age- and sex-matched animals, ensuring that observed effects were attributable to the treatment rather than biological variability, in line with best practices for preclinical modeling of ASD [140].

Dysbiosis can lead to increased intestinal permeability and systemic inflammation, contributing to the activation of microglia and reactive astrocytes in the brain. For example, microbiota impacts microglial molecular functions by promoting the expression of the complement signaling pathway and the synaptic remodeling factor C1q, both of which are essential for brain development and immune responses. These influences may contribute to neurodevelopmental conditions like ASD by affecting synaptic pruning and neuroinflammation [141].

**Fig. 2:**
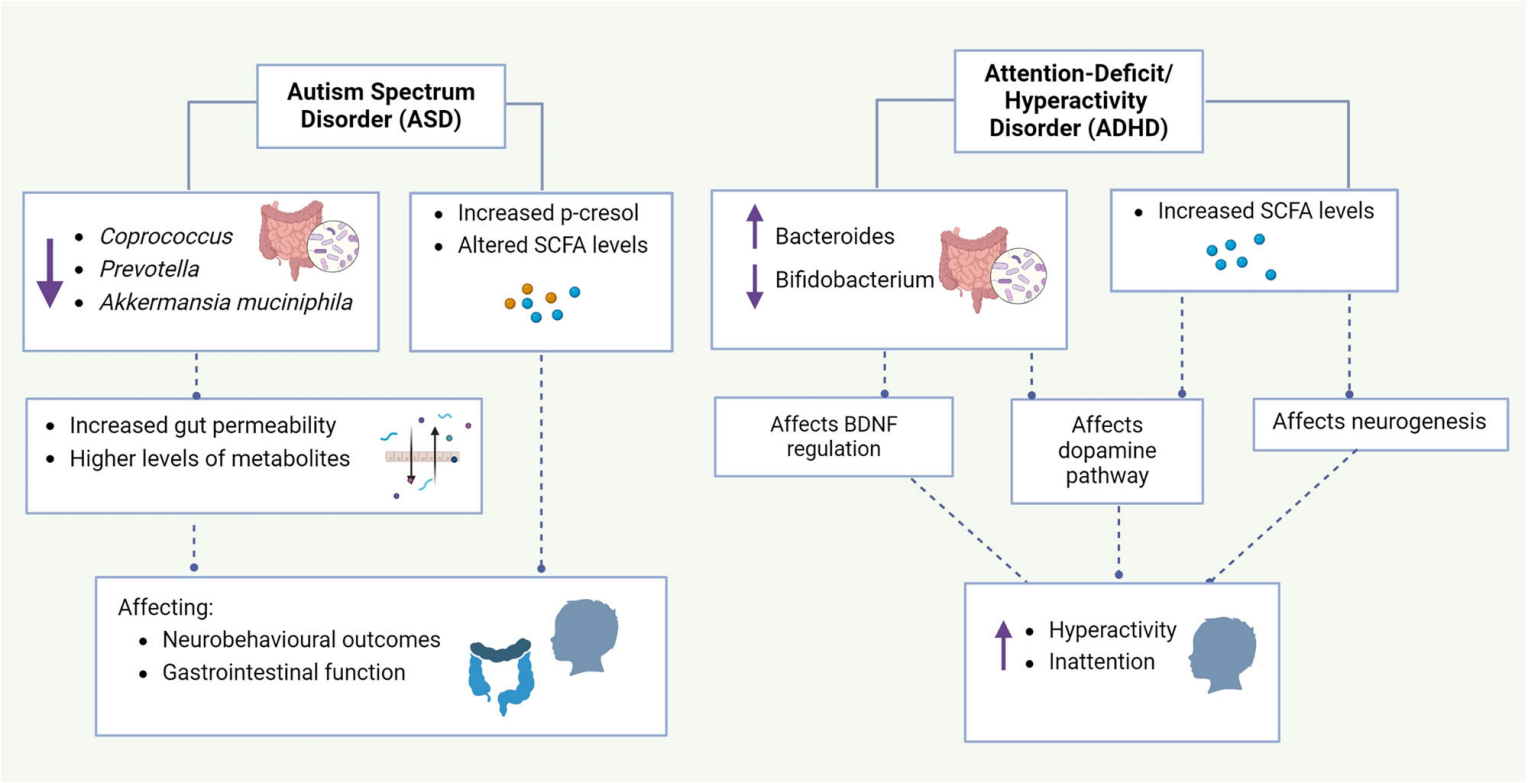
Impact of the gut microbiota on ASD and ADHD. The GM significantly influences neurodevelopmental disorders such as ASD and ADHD. Individuals with ASD exhibit reduced diversity in the gut microbiota, with decreased levels of *Coprococcus, Prevotella*, and *Akkermansia muciniphila*, which are linked to increased gut permeability and increased levels of metabolites, affecting neurobehavioral outcomes. Alterations in bacterial metabolites, such as increased p-cresol and altered SCFA levels, are correlated with autism symptom severity, influencing social behaviors and gastrointestinal function. In ADHD patients, changes in the GM composition, including increases in Bacteroides and decreases in Bifidobacterium, impact dopamine production and BDNF regulation, contributing to symptoms of inattention and hyperactivity. Elevated SCFA levels influence neurogenesis and dopamine pathways, potentially impacting attention and hyperactivity symptoms.

In summary, the intestinal microbiota of individuals with ASD differs from that of healthy individuals. Certain bacteria, such as *Akkermansia muciniphila* and *Bifidobacterium spp*., are relatively low in abundance in the fecal samples of children with ASD. Additionally, the levels of metabolites such as SCFAs are altered in individuals with ASD, with changes associated with specific bacterial groups. Elevated levels of microbial metabolites such as p-cresol have been observed in fecal samples from children with ASD, and their urinary levels correlate with autism symptom severity. However, recent findings suggest that microbiome differences in ASD may largely reflect dietary preferences rather than a causal role in ASD [142]. These findings caution against assuming a direct role of the microbiome in driving ASD and highlight the need for further research to distinguish between microbiome changes due to diet and those directly contributing to ASD. Overall, further research is needed to elucidate the mechanisms by which alterations in the gut microbiota contribute to ASD and to explore potential therapeutic interventions targeting the gut microbiome to improve symptoms and quality of life in individuals with ASD (Fig. 2).

#### Attention-deficit/hyperactivity disorder (ADHD)

ADHD is a neurodevelopmental disorder that affects approximately 5% of children and 2.5% of adults. ADHD presents as a consistent behavioral pattern of inattention and/or hyperactivity coupled with impulsivity [128, 143]. Reduced activity of brain-derived neurotrophic factor (BDNF), an important factor in the growth, development, and survival of neurons, in the midbrain may lead to dysfunction in dopaminergic pathways, potentially contributing to ADHD symptoms. Additionally, bacterial metabolites such as SCFAs have been shown to influence neurogenesis by triggering the release of BDNF [144, 145]. In ADHD patients, there was a slight increase in *Bifidobacterium* levels alongside a significant increase in 16S-based bacterial gene functionality, which encodes an enzyme called cyclohexadienyl dehydratase. The production of phenylalanine, a precursor to dopamine, depends on this enzyme [114]. A case-control study examining gut microbiota and dietary patterns in children with attention-deficit/hyperactivity disorder (ADHD) revealed that children with ADHD exhibited increased relative abundances of *Bacteroides ovatus*, *Bacteroides uniformis*, and *Sutterella stercoricanis* compared to healthy controls. Notably, the study reported that the ADHD and control groups were matched for age and sex, ensuring that observed differences in gut microbiota composition were not confounded by these variables. Additionally, *S. stercoricanis* demonstrated significant associations with dietary intake of dairy, nuts/seeds/legumes, ferritin, and magnesium, suggesting a potential link between diet, gut microbiota, and ADHD symptoms [146]. ADHD risk has been associated with a reduced relative abundance of *Bifidobacterium* in fecal samples during early-life *Streptococcus* infection [147].

Taken together, these findings show that microbial composition could influence the development and expression of ADHD symptoms. Potential mechanisms may involve particular bacterial species, such as *Bifidobacterium* and *Bacteroides*, affecting neurotransmitter pathways, including dopamine production. Moreover, the importance of bacterial metabolites, such as SCFAs, has been suggested to regulate neurogenesis and BDNF levels, which are pivotal for neuronal development and function (Fig. 2).

### Neuropsychiatric disorders

There is increasing evidence linking GM dysbiosis to the pathophysiological processes underlying neuropsychiatric diseases. However, it is still mostly unclear how exactly the gut microbiota contributes to these disorders. Moreover, both clinical and preclinical evidence has shown that microbiota perturbations can partially alleviate psychiatric symptoms caused by treatment with probiotics [148]. Therefore, in this part of the review, we discuss evidence supporting the role of the GM in neuropsychiatric disorders and the mechanisms underlying its contribution to these disorders (Table 1).

#### Depression

More than 35 million individuals worldwide are affected by major depressive disorder (MDD), which is characterized by long-lasting low moods influenced by both genetic factors and environmental elements [149]. Although peripheral proinflammatory cytokine levels and activated inflammasomes are frequently high in MDD patients, there is no evidence that these individuals’ peripheral cells are directly trafficked into the brain [150]. The GM can modulate the kynurenine pathway. Imbalances in the kynurenine pathway have been associated with inflammation and neuroinflammation, which are implicated in the pathophysiology of depression [151].

Caspase-1 is a protease that plays a crucial role in the inflammatory process by activating pro-inflammatory cytokines such as IL-1β and IL-18 [152]. Blocking caspase-1 is well known to reduce both inflammation and anxiety-like behaviors but has also been shown to influence the composition of the gut microbiota. Mice treated with anti-caspase-1 antibodies exhibit elevated levels of *Blautia spp*. and *Akkermansia spp*., which are associated with the activation of Foxp3 in regulatory T cells and the inhibition of pathways mediated by IL-1β and IL-6, leading to reduced inflammation [116–118]. It suggests that targeting caspase-1 could be a potential therapeutic strategy for modulating gut microbiota to promote the activation of regulatory T cells and suppress pro-inflammatory cytokine pathways. Mice exposed to ampicillin developed both colitis and anxiety, with a notable increase in the population of *Proteobacteria*, specifically *Klebsiella oxytoca*. *L. reuteri* subsequently results in reduced inflammation in the CNS and the alleviation of symptoms associated with anxiety [153]. G protein-coupled receptors (GPCRs), particularly GPR41 and GPR43 on gut epithelial cells, can be activated by SCFAs. This activation then initiates the mitogen-activated protein kinase (MAPK) pathway, leading to tissue inflammation in mice [154]. Dysbiosis is linked to both the NLRP3 and the NLRP6 inflammasome, and mice lacking NLRP3 or NLRP6 exhibit aggravated colitis. IL-18, which is released when the NLRP3 inflammasome in the gut epithelium is activated by elevated amounts of SCFAs produced by the GM, has a protective effect against colitis and contributes to gut homeostasis [155]. Microbiota homeostasis is also facilitated by NLRP6, and its deficiency in mouse colonic epithelial cells leads to reduced levels of IL-18 and dysbiosis through changes in the GM composition [156]. Notably, SCFAs regulate microglial maturation and function suggesting that disturbances in NLRP3/SCFA signaling in the gut may contribute to aberrant microglial activation in the brain [41]. Thus, gut dysbiosis and inflammasome dysfunction may link intestinal and neuroimmune pathways, influencing microglial responses and potentially contributing to neurodevelopmental or neuroinflammatory disorders.

**Fig. 3:**
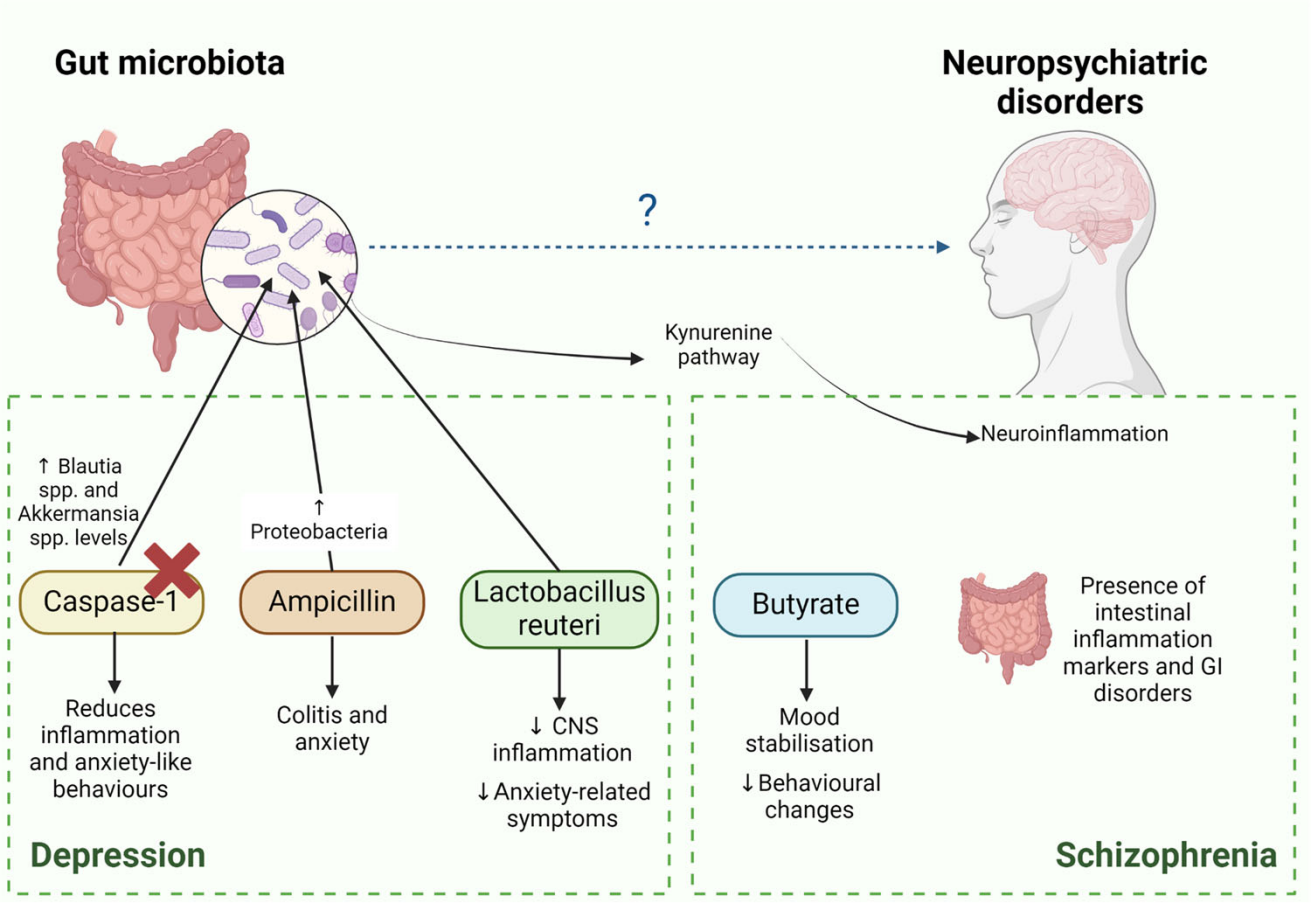
Influence of the gut microbiota on neuropsychiatric disorders. Alterations in the gut microbiota contribute to depression through mechanisms involving inflammation and the kynurenine pathway. Dysbiosis influences the availability of tryptophan for serotonin synthesis and impacts SCFA levels, which are linked to mood regulation and depressive symptoms. Blockade of caspase-1 has been found to affect the composition of the gut microbiota and reduce inflammation and anxiety-like behavior. Schizophrenic patients have distinct microbiota compositions with reduced butyrate-producing bacteria, such as *Blautia* and *Roseburia*. Gut‒brain interactions involving the kynurenine pathway contribute to neuroinflammation, with microbial metabolites influencing oxidative stress and excitotoxicity, which are associated with schizophrenia symptoms.

Tryptophan is an essential amino acid that serves as a precursor for the synthesis of serotonin, a neurotransmitter known to play a crucial role in regulating mood and emotions. Gut microorganisms can convert tryptophan into substantial metabolites such as melatonin, serotonin, and indole and therefore influence the availability of tryptophan for serotonin synthesis [151]. *Clostridium and Proteus*, two genera of the GM, can generate metabolites such as indole and tryptophan that can pass through the BBB and act as neuroprotectants against reactive oxygen species (ROS) [157]. Dysbiosis can lead to alterations in tryptophan metabolism, potentially impacting serotonin levels and contributing to the development or exacerbation of depressive symptoms (Fig. 3).

In summary, alterations in the composition of the GM can influence inflammation, the gut‒brain axis, and ultimately mood regulation in individuals with depression. Therefore, the potential therapeutic implications of targeting the gut microbiota may modulate inflammation and improve depressive symptoms.

#### Schizophrenia

Schizophrenia is a devastating psychiatric disorder characterized by negative symptoms such as apathy, slowness and withdrawal, as well as positive symptoms such as aberrant flow of thoughts, delusions and hallucinations [158]. Schizophrenic patients with both recent and non-recent onset schizophrenia exhibited significantly elevated levels of anti-*Saccharomyces cerevisiae* antibodies (ASCA) compared to non-psychiatric controls. The study also noted that these elevated ASCA levels were particularly evident in antipsychotic-naïve individuals, suggesting that the observed GI inflammation was not solely a consequence of medication effects. These findings highlight a potential link between intestinal inflammation and schizophrenia, indicating the importance of considering GI health in the context of psychiatric disorders [159]. This aligns with the observation that gastrointestinal disorders frequently present as coexisting conditions with schizophrenia.

Recent research has revealed variations in the GM between individuals with schizophrenia and those without symptoms. These findings suggest a potential link between specific bacteria in the microbiome and symptoms associated with schizophrenia, possibly through interactions involving gut–brain lipid and amino acid metabolic pathways [160]. Butyrate compounds have the potential to stabilize mood. Research indicates that sodium butyrate, a HDAC inhibitor, can reverse changes in neurotrophins, the TCA cycle, oxidative stress, the mitochondrial respiratory chain and behavioral changes in amphetamine-induced animal models of depression and mania [161]. In individuals with schizophrenia, the prevalence of gut microbiota capable of producing butyrate, such as *Blautia*, *Roseburia*, and *Coprococcus*, is lower than that in healthy individuals [162].

The GM also plays a role in the kynurenine pathway, which is related to the process of neuroinflammation in patients with schizophrenia [163]. The kynurenine pathway in the CNS involves the conversion of kynurenine to neurotoxic quinolinic acid in microglia, contributing to oxidative stress and excitotoxicity. Conversely, in astrocytes, kynurenine converts to neuroprotective kynurenic acid, which exhibits anti-inflammatory and immunosuppressive properties [164]. Fecal microbiota transplantation from schizophrenic patients to mice treated with antibiotics resulted in behavioral abnormalities and elevated kynurenine pathway activity in specific brain regions compared with those in mice receiving feces from healthy controls [165]. In mice, oral *Bifidobacterium* supplementation showed a therapeutic effect on resilience to social defeat stress, whereas *Lactobacillus reuteri* reduced despair-like behavior in a chronic mild stress model by suppressing host kynurenine metabolism and increasing kynurenine levels [166] (Fig. 3).

Ultimately, these findings suggest a link between the gut microbiome, the kynurenine pathway, and schizophrenia, present opportunities for innovative therapeutic approaches and provide a deeper understanding of the complex interactions shaping mental health.

### Neurodegenerative disorders

Neurodegenerative diseases (NDs) are increasingly prevalent and significant contributors to mortality and morbidity globally, especially in the elderly population. Accurate diagnosis is crucial because it enhances reliable prognosis and frequently directs precise treatment and management strategies [167]. The onset, progression, and severity of neurodegenerative diseases are influenced by risk factors such as genetic predispositions and lifelong exposure to environmental factors [168]. During dysbiosis, signals are transmitted to the brain through the gut‒brain axis, resulting in disturbed energy metabolism and increased inflammation, oxidative stress, and cell degeneration [169]. These signals during dysbiosis contribute to the pathological progression of various neurological disorders, especially neurodegeneration [170], and elderly patients with neurodegenerative disorders have a striking change in GM composition [171]. Recent research has emphasized the significant role of the microbiota in influencing the maturation and function of microglia, which are recognized as among the initial contributors to the process of neurodegeneration [65].

In this review, we focus on the role of the GM composition and its metabolites in neurodegenerative disorders, including PD and AD. Moreover, in this section, we discuss the associations among specific GM species, metabolites, and neurodegenerative processes, such as amyloid aggregation, neuroinflammation, and neuronal death, in PD and AD. We also address the potential therapeutic implications of modulating the GM to mitigate neurodegenerative changes and improve outcomes for individuals affected by these conditions.

### Alzheimer’s disease (AD)

AD is one of the predominant types of dementia and is clinically characterized by a progressive decline in cognitive function and memory problems [172]. Studies suggest a link between cognitive impairment and brain amyloidosis with increased proinflammatory *Escherichia/Shigella* and decreased anti-inflammatory *Eubacterium rectale* in the gut [173, 174]. The beneficial effects of SCFAs include reducing Aβ aggregation [175], and *Clostridium butyricum*, which generates the SCFA butyrate, was shown to have anti-neuroinflammatory effects and be protective against AD [86]. GF-AD mice show reduced Aβ plaque load and low SCFA levels, while SCFA supplementation restores or worsens plaque accumulation, suggesting SCFAs may promote Aβ pathology in AD [176]. Both *Bacteroides vulgatus* and *Campylobacter jejuni* can affect cognitive function in individuals diagnosed with Alzheimer’s disease [177]. Moreover, probiotic strains like *Lactobacillus* and *Bifidobacterium* have been shown to improve cognitive function and memory in both animal models and human subjects with AD and mild cognitive impairment [178].

Curli amyloids, secreted by *Enterobacteriaceae*, resemble human amyloids. They have structural and physical similarities and can trigger caspase 1 via NLRP3 inflammasome activation [179, 180]. Treating rats with Curli-producing bacteria on a regular basis results in increased production of cytokines, microglia, and astrocytes [181].

**Fig. 4:**
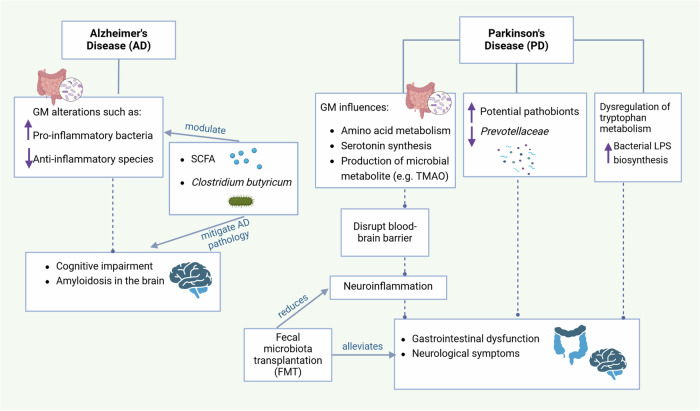
Influence of the gut microbiota on neurodegenerative disorders. The GM significantly influences neurodegenerative disorders such as AD and PD. In AD, a decrease in anti-inflammatory species and an increase in proinflammatory bacteria may be linked to amyloidosis and cognitive decline. Certain bacteria, such as *Clostridium butyricum*, and microbial metabolites, including SCFAs, have demonstrated therapeutic potential in modulating the gut microbiota to reduce AD pathogenesis. PD has frequent comorbidities with gastrointestinal issues and neurological symptoms and is associated with reductions in cellulose-degrading bacteria and neuroprotective *Prevotellaceae*, as well as elevations in potentially pathogenic bacteria. The GM influences acid metabolism, serotonin synthesis, and the formation of microbial compounds such as trimethylamine N-oxide (TMAO), disrupting the blood‒brain barrier and contributing to neurological inflammation. Dysregulation of tryptophan metabolism and increased bacterial LPS production have also been linked to PD pathogenesis. FMT has emerged as a viable strategy for preventing PD by lowering neuroinflammation and altering signaling pathways.

LPS derived from *Bacteroides fragilis* (BF-LPS) can affect the CNS after it crosses the intestinal barrier. While it may not directly cross the BBB, it can still influence the CNS through systemic inflammation, potential disruption of the BBB, and activation of neural pathways that communicate inflammatory states to the brain. These processes contribute to neuroinflammation such as activation of microglia and the neurodegenerative changes observed in conditions such as AD [182] (Fig. 4).

In summary, alterations in the GM composition, such as increased proinflammatory bacteria and decreased anti-inflammatory species, may contribute to cognitive impairment and amyloidosis in the brain. Moreover, the beneficial effects of certain microbial metabolites, such as SCFAs, and specific bacteria, such as *Clostridium butyricum*, indicates the therapeutic potential of modulating the gut microbiome to mitigate AD pathology. Additionally, the involvement of microbial amyloids and lipopolysaccharides in triggering neuroinflammatory cascades further highlights the intricate interplay between gut microbes and CNS homeostasis, potentially influencing the neurodegenerative changes characteristic of AD. These findings suggest an intricate relationship between amyloid formations, dysbiosis of the gut microbiota, and neuroinflammation, highlight possible causes for the progression of AD and point to potential novel treatment approaches.

### Parkinson’s disease (PD)

PD is characterized by the progressive accumulation of α-synuclein in the brain, leading to neuroinflammation and neuronal death, and is the most prevalent neurodegenerative disease after AD [183, 184]. Clinical studies have highlighted similarities between gastrointestinal problems and neurological symptoms in PD patients, indicating common comorbidities and correlations between PD-related neurological symptoms and gastrointestinal dysfunction. Dopamine (DA), a brain hormone, is produced by dopaminergic neurons in the substantia nigra (SN). In PD, there is a progressive loss of these neurons and a reduction in dopamine levels in the substantia nigra pars compacta [185]. The gut microbiome affects dopamine levels in brain regions such as the frontal cortex and striatum in rodent models. Dysbiosis of the gut microbiota contributes to PD development, and FMT has shown promise in protecting PD mice by reducing neuroinflammation and modulating signaling pathways [8]. In mice with elevated levels of αSyn, a protein implicated in PD, the GI microbiota exacerbates motor deficits, microglial activation, and α-Syn pathology [186].

Individuals with PD exhibit significant alterations in gut microbiota composition, characterized by a reduction in cellulose-degrading bacteria such as *Ruminococcus*, *Faecalibacterium*, and *Blautia*, alongside an increase in potential pathobionts including *Enterococcus*, *Streptococcus*, *Proteus*, *Escherichia*, and *Shigella*. These microbial shifts have been observed in studies comparing PD patients to age- and sex-matched healthy controls, underscoring the importance of controlling for these variables when assessing gut microbiota differences associated with PD [187]. Several studies have consistently indicated a decreased relative abundance of *Prevotellaceae* in PD patients. *Prevotellaceae* has neuroprotective effects by producing SCFAs [188–190], and a lower *Prevotellaceae* abundance may lead to the accumulation of α-synuclein, which is a neuropathological hallmark in PD patients [191]. Fecal samples from healthy people had a greater abundance of bacteria with the ability to produce butyrate than those from PD patients, who had a reduced abundance of *Faecalibacterium* spp. and *Lachnospiraceae* family members [192–194]. Serum SCFAs, especially propionic acid, are reduced in PD patients and correlate with motor and cognitive symptoms. This suggests gut-derived SCFAs may influence PD via circulation, with propionic acid showing potential for symptom relief pending clinical validation [195]. PD progression has also been linked to a decline in SCFA-producing genera such as *Fusicatenibacter, Faecalibacterium*, and *Blautia*, suggesting gut dysbiosis may play a contributing role in disease advancement [196]. The distribution of aromatic and branched-chain amino acids in the gut is influenced by the GM, which is important for amino acid metabolism. Therefore, altered gut microbiota may be related to the plasma amino acid imbalances observed in PD patients [197]. Trimethylamine N-oxide (TMAO), derived from gut microbes and certain dietary components, can disrupt the blood‒brain barrier, activate the NLRP3 inflammasome, and induce α-syn folding, potentially contributing to neuroinflammation and PD. Thus, limiting microbiota-produced TMAO deserves further investigation as a potential complement to existing therapies.

The synthesis of serotonin relies on tryptophan. When other pathways for tryptophan metabolism become more active, it leads to increased consumption of tryptophan. This heightened consumption, in turn, diminishes the synthesis of serotonin. Such serotonin deficiency has been linked to the underlying processes of PD [198]. Certain types of bacteria, specifically *Eubacteria*, have been connected to reduced expression of genes required for the breakdown of 5-dihydro-4-deoxy-D-glucuronate, along with two additional pathways related to tryptophan metabolism that lead to increased conversion of formate, as reported in a metabolomics study of PD patients [199, 200].

The bacterial genes responsible for LPS biosynthesis presented elevated activity in both mucosal and fecal samples taken from patients with PD. Interestingly, the levels of serum lipopolysaccharide-binding protein (LBP) are lower in PD patients than in controls, particularly when the integrity of the intestinal mucosa is intact [201]. In PD fecal microbiomes, several genes associated with metabolism, such as metabolic pathways and ABC transporters, presented significantly reduced expression levels, whereas genes related to LPS biosynthesis and type III bacterial secretion systems were notably increased [202]. A relatively high level of LPS, resulting from increased intestinal permeability, can lead to the excessive expression and aggregation of αSyn. This phenomenon underscores the potential role of intestinal dysbiosis in PD pathogenesis, as supported by findings from colon biopsies of PD patients, which revealed increased expression of toll-like receptor 4 (TLR4), CD3 + T lymphocytes, and various cytokines associated with intestinal dysbiosis [203, 204].

In summary, PD shares common comorbidities and correlations between gastrointestinal problems and neurological symptoms. FMT has emerged as a promising approach for protecting against PD by reducing neuroinflammation and modulating signaling pathways. Specific alterations in GF composition, such as reductions in cellulose-degrading bacteria and neuroprotective *Prevotellaceae*, alongside increases in potential pathobionts, are observed in individuals with PD. Furthermore, the gut microbiota influences amino acid metabolism, serotonin synthesis, and the production of microbial metabolites such as TMAO, which can disrupt the BBB and contribute to neuroinflammation. Dysregulation of tryptophan metabolism and increased bacterial LPS biosynthetic activity are also implicated in PD pathogenesis, suggesting the intricate role of the gut microbiota in disease progression. These findings highlight the potential therapeutic implications of targeting the gut microbiome in PD management (Fig. 4).

#### Conclusion

The gut microbiota (GM) plays a crucial role in influencing brain function and neurological health through the gut‒brain axis. Dysbiosis in the gut can lead to disturbances in energy metabolism, increased inflammation, oxidative stress, and impaired brain plasticity through microbiota metabolites, neurotransmitter production, and the modulation of microglial function. Understanding the relationship between the gut microbiota and brain cells opens new possibilities for therapeutic interventions in various neurological disorders.
